# Answer to J. Y. on Mr. Brown's Observ. on Zoonomia

**Published:** 1800-02

**Authors:** 


### *To the Editors of the Medical and Phyfical Journal.*

Gentlemen,

In the eighth Number of your valuable Mifcellany, the author of "Obfervations on Zoonomia," is, by one of your correfpondents, accufed of mifreprefentation. Having been much delighted with Dr. Darwin's Theory, I have paid confiderable attention to the arguments of his controverfialift; and, whatever his other deficiencies may be, I have found no reafon to charge him with mifconception. I therefore take the liberty of addreffing a few words to your correfpondent; and, I truft, your candour will confider the admiffion of the accufation, as a fufficient reafon for admitting the defence.

The following paffage is that which J. Y. adduces, as the inftance of miftatement. "The embryon, according to Dr. Darwin, is a fimple filament, without fenforial power, or the means of producing it·" Dr. Darwin himself terms the filament *simple*, p. 496; and though, in the passages quoted by your correspondent, it is said to be "a living filament, *with certain capabilities of irritation, sensation, volition, and association*," the periphrasis seems to shew, that essential, rather than derived energy, is ascribed to the filament. This supposition is indeed inconsistent with the assertion, that life consists in the motions of sensorial power. But Dr. Darwin had a separate theory to support; he saw that a small quantity of a fluid liable to quick exhaustion, would be of little service to his embryon; and he therefore chose to ascribe to it permanent activity. He does not indeed say, that it has not the means of producing sensorial power; but Mr. Brown, afterwards, shews this to be the necessary result of the principles of Zoonomia. The conciseness which Mr. Brown has used in the section on sensorial power, I suppose, proceeded from the desire of avoiding the unpleasantness of repetition, as the subject is afterwards fully discussed by him in the section on generation.

As it is to the theory of generation the supposed misstatement relates, the objector should have thought it necessary to examine that part of the work, before advancing his charge. Had he consulted p. 435 of the Observations, he would have found, that Mr. Brown is not merely innocent of the alleged misrepresentation, but has himself quoted, and fully considered, the principal passage recommended to his notice by your correspondent. The passage can be understood only in two senses: It either states the existence of sensorial power in the primordial filament, or of energies of life, independent of sensorial power. The latter, however inconsistent with the principles of Zoonomia, seems to be the real sense in which it is used by Dr. Darwin; but Mr. Brown has considered it in both senses; and, it might have been thought, is thus doubly fortified against the charge of misrepresentation.

The following passage of the Observations, p. 434, will therefore, I trust, be considered by J. Y. as a sufficient answer.

"The process of generation is said to consist in the secretion by the male of a living filament, and by the female of a nutritive fluid, which stimulates the filament to absorb certain particles, and thus to add to its bulk." "At the earliest period of its existence, the embryon, as secreted from the blood of the male, would seem to consist of a living filament, with certain capabilities of irritation, sensation, volition, and association." Zoon. p. 484. To say that the filament is living, and that it possesses these powers, is to say, that it possesses sensorial power, which is considered by Dr. Darwin as the source of animation. But the small quantity of sensorial power, which we must suppose it to possess, will be exhausted in a short time, by the continual irritation of the particles around it; and the embryon will thus be deprived of life, almost as soon as it is separated from the male.

It will, perhaps, be asserted, though the assertion cannot be reconciled with the division on which Zoonomia is founded, that the four sensorial faculties are possessed by the filament, without reference to its sensorial power, and that it is thus essentially endued with life. It is not sufficient, however, that the filament originally secreted, possess the four faculties of animation. The parts joined by accretion, must also possess them; for the original filament will be surrounded by these, long before the complete formation of the brain and spinal marrow. If, therefore, the whole frame be susceptible of irritation, sensation, volition, and association, the brain and spinal marrow are wholly useless. Dr. Darwin will find it difficult to explain those phænomena, which he ascribes to the absence of irritability, or voluntarity; and death will be impossible."

